# Detecting Statistically Significant Common Insertion Sites in Retroviral Insertional Mutagenesis Screens

**DOI:** 10.1371/journal.pcbi.0020166

**Published:** 2006-12-08

**Authors:** Jeroen de Ridder, Anthony Uren, Jaap Kool, Marcel Reinders, Lodewyk Wessels

**Affiliations:** 1 Information and Communication Theory Group, Faculty of Electrical Engineering, Mathematics, and Computer Science, Delft University of Technology, Delft, The Netherlands; 2 Division of Molecular Biology, The Netherlands Cancer Institute, Amsterdam, The Netherlands; 3 Division of Molecular Genetics, The Netherlands Cancer Institute, Amsterdam, The Netherlands; University of Pennsylvania School of Medicine, United States of America

## Abstract

Retroviral insertional mutagenesis screens, which identify genes involved in tumor development in mice, have yielded a substantial number of retroviral integration sites, and this number is expected to grow substantially due to the introduction of high-throughput screening techniques. The data of various retroviral insertional mutagenesis screens are compiled in the publicly available Retroviral Tagged Cancer Gene Database (RTCGD). Integrally analyzing these screens for the presence of common insertion sites (CISs, i.e., regions in the genome that have been hit by viral insertions in multiple independent tumors significantly more than expected by chance) requires an approach that corrects for the increased probability of finding false CISs as the amount of available data increases. Moreover, significance estimates of CISs should be established taking into account both the noise, arising from the random nature of the insertion process, as well as the bias, stemming from preferential insertion sites present in the genome and the data retrieval methodology. We introduce a framework, the kernel convolution (KC) framework, to find CISs in a noisy and biased environment using a predefined significance level while controlling the family-wise error (FWE) (the probability of detecting false CISs). Where previous methods use one, two, or three predetermined fixed scales, our method is capable of operating at any biologically relevant scale. This creates the possibility to analyze the CISs in a scale space by varying the width of the CISs, providing new insights in the behavior of CISs across multiple scales. Our method also features the possibility of including models for background bias. Using simulated data, we evaluate the KC framework using three kernel functions, the Gaussian, triangular, and rectangular kernel function. We applied the Gaussian KC to the data from the combined set of screens in the RTCGD and found that 53% of the CISs do not reach the significance threshold in this combined setting. Still, with the FWE under control, application of our method resulted in the discovery of eight novel CISs, which each have a probability less than 5% of being false detections.

## Synopsis

A potent method for the identification of novel cancer genes is retroviral insertional mutagenesis. Mice infected with slow transforming retroviruses develop tumors because the virus inserts randomly in their genome and mutates cancer genes. The regions in the genome that are mutated in multiple independent tumors are likely to contain genes involved in tumorigenesis. As the size of these datasets increases, conventional methods to detect these so-called common insertion sites (CISs) no longer suffice, and an approach is required that can control the error independent of the dataset size. The authors introduce a framework that uses a technique called kernel density estimation to find the regions in the genome that show a significant increase in insertion density. This method is implemented over a range of scales, allowing the data to be evaluated at any relevant scale. The authors demonstrate that the framework is capable of compensating for the inherent biases in the data, such as preference for retroviruses to insert near transcriptional start sites. By better balancing the error, they are able to show that from the 361 published CISs, 150 can be identified that have a low probability of being a false detection. In addition, they discover eight novel CISs.

## Introduction

### Retroviral Tagging

In retroviral insertional mutagenesis experiments, genes involved in the development of cancer are identified by determining the loci of viral insertions from tumors induced by retroviruses in mice [1,2]. After infecting a host cell, the retrovirus inserts its own DNA into the host cell's genome, mutating the host cell's DNA in the process. The mutation may alter the expression of genes in the vicinity of the insertion or, when inserted within a gene, alter the gene product. When the affected gene is a cancer gene (either a proto-oncogene or a tumor suppressor gene), activation of the proto-oncogene or inactivation of the tumor-suppressor gene can cause uncontrolled proliferation (cell division) of cells. Eventually this may give rise to tumors. Throughout this text, these cancer-causing insertions are referred to as oncogenic insertions.

A tumor develops when an accumulation of oncogenic insertions causes uncontrolled proliferation of a cell. As a result, the tumor tissue contains many copies of the cell bearing the oncogenic insertions that induced the proliferation, but only a few copies of cells carrying non-oncogenic (random, background) insertions. Consequently, when the DNA of the tumor is analyzed, one will encounter the insertion that induced proliferation in larger proportions than insertions that do not. Regions in the genome that are found to carry insertions in multiple independent tumors are called common insertion sites (CISs). As a result, the locations of the CISs are highly correlated with the location of genes involved in tumor development. Cloning the flanking sequences of the inserted virus to determine the insertion loci, and analyzing these data to find significant CISs, therefore enable the discovery of new candidate cancer genes. This is summarized in Figure S1.

### The Data

Over the last few years an extensive amount of insertional mutagenesis data has been published [3–10]. These data have been compiled in the Retroviral Tagged Cancer Gene Database (RTCGD) [11] (http://RTCGD.ncifcrf.gov), and contains approximately 4,000 insertions (accessed November 2005). The vast majority of these insertions have been acquired in 20 different screens that each analyzed the insertions using their own definition of a CIS, which sometimes includes manual curation.

Due to noise in the data, not all insertions are informative. In the idealized case, oncogenic insertions are present in every tumor cell (since these cells are all copies of the cell with the initial oncogenic insertion that induced the tumor), whereas background insertions are only present in a small proportion of the tumor cells. Although this implies that the probability of finding a non-oncogenic insertion is far lower than for an oncogenic insertion, it may still occur that a non-oncogenic insertion is found. This results in non-informative insertions, the noise. Moreover, when a non-oncogenic insertion happens early in the tumor development phase, i.e., co-occurs in the same cell with one or more oncogenic insertions, there will also be many copies of this non-oncogenic insertion in the final tumor. Consequently, the probability of mapping this insertion will increase dramatically. This phenomenon is called *piggy-backing*. Due to piggy-backing, it is required that an insertion at a certain locus occurs in more than one tumor, since this greatly reduces the probability of finding a CIS that is not causal for the tumor.

### Modeling Common Insertion Sites

**Figure 1:**
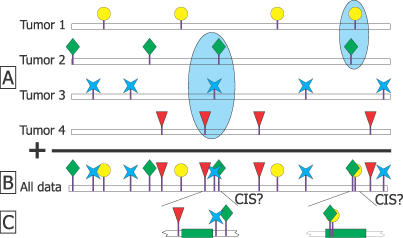
Schematic View of Insertion Data (A) Schematic view of the mapped data of four tumors. Significance is determined by the number of tumors which contain insertions in a particular region. The geometric symbols represent the insertions and are given a different shape for each tumor. The blue regions indicate possible CISs. (B) When considering a broad region, the number of insertions one would expect to have occurred by chance is higher, and hence the regions need to be hit in more independent tumors than for narrow regions before significance is reached. (C) Genes (indicated by the green bars) may be affected from various loci around or within the gene, and there does not exist one distance over which viral inserts act on their targets.

A CIS is defined as a region in the genome that has been hit by viral insertions in multiple independent tumors significantly more frequently than expected by chance (schematically illustrated in Figure 1). Ideally, CISs are identified because insertions in this region induce an oncogenic mutation that affects nearby cancer genes. Cancer genes may, however, be affected from various regions around or within the gene. It is yet unclear what determines the width of these regions, but it is certain that there does not exist one such width applicable to all cancer genes. This biological variance should be accounted for when evaluating the statistical significance of CISs.

For the analysis of the individual screens in the RTCGD, previous methods used one, two, or three windows of fixed size, and obtained an estimate of the number of false CISs by using Monte Carlo simulation [9] or the Poisson distribution [8]. When the amount of data (insertion sites) increases, as is expected in the near future, these analyses will suffer from an increase in the probability of finding false CISs. To reduce the number of false detections, the window size has to be decreased, such that the method performs at the desired error level. This type of error control is undesirable, since this results in a mismatch between the new, decreased, window size, and the biologically relevant window size (the scale of the putative CIS), and hence may cause CISs to be missed. Therefore, methods for detecting CISs should be capable of keeping control of the probability of detecting false CISs, independent of the scale of the putative CIS.

The definition of a CIS depends on some expectation of the insertion rate associated with non-CIS regions. For this reason, we have to make assumptions about the background insertion distribution, i.e., the distribution of insertions under the assumption that there is no proliferative selection. In current methods, this distribution is assumed to be uniform, i.e., viral inserts show no preference for specific regions in the genome. Various authors suggest, however, that viral inserts do show local biases [12–15]. Specifically, it is suggested that murine leukemia virus (MLV) favors integration near transcription start sites (TSSs) due to local recognition of genomic features [14,15]. When the goal is to identify the regions that are involved in the tumor development process, these so called hot spots should be corrected for [16].

Summarizing, we state that, for the detection of CISs in retroviral insertional mutagenesis data, a framework is needed that 1) evaluates significance at any desired (biologically relevant) scale, 2) does so while keeping control of the error (since in the near future a significant increase in these data is expected), and 3) provides the possibility of including a background distribution, enabling compensation for the background bias. In this study, we propose a kernel convolution (KC) framework that meets the criteria outlined above. We apply this framework to the data of all the screens in the RTCGD combined, as if they originated from one screen. This gives us the opportunity to evaluate the method for large amounts of data. Indeed, the method rejects 53% of the CISs that do not reach the significance level in the combined set of screens. While keeping the family-wise error (FWE) under control, the method still revealed eight new CISs that are significant across the different screens. In addition, we provide the nearby putative target genes that may play a role in oncogenesis. Due to its generality, the method can be applied to other types of high-throughput genome-wide data, too; for example, to copy number aberration data or data from insertional mutagenesis screens using transposons [17].

## Results

### The Kernel Convolution Framework

**Figure 2:**
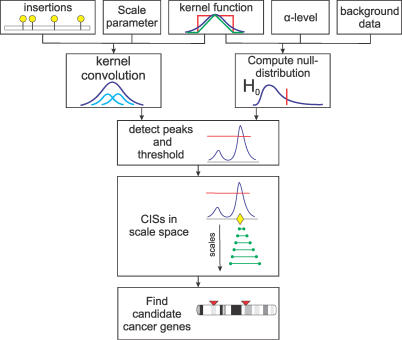
Schematic Depiction of the Kernel Convolution Framework The insertions are convolved with a kernel function with a width determined by the scale parameter. In principle any kernel function can be used, but the Gaussian kernel function is depicted. The significance of the peaks is evaluated using a null-distribution computed by means of a random permutation of the data. This is done for a range of scale parameters to obtain the CISs in the scale space.

The steps involved in the application of the KC framework can be summarized as follows (see Figure 2, for details see the Methods section). A kernel function is positioned at every insertion in the data. For any position in the genome, an estimate of the number of insertions can be obtained by summing all the kernel functions. Applying a KC effectively smoothes the observed insertion in a region around this insertion. This alleviates the problems associated with the inherent data sparseness, since we use the observed insertion to infer information about its direct neighborhood. Insertions occurring in each others vicinity will produce a higher peak in the estimate of the number of insertions. This models the fact that these insertions may all have an effect on a nearby cancer gene. The kernel width, which controls the smoothness of the estimate of the number of insertions, can be seen as a scale parameter. By varying the scale parameter, CISs of varying widths can be detected. The peaks in the estimate of the number of insertions are indicative for the location of putative CISs. Peaks are significant when they exceed an amplitude threshold. This threshold is determined based on a user-defined *α*-level, and the empirical null-distribution of peak heights, which is obtained by random permutation experiments. In addition to a uniform background distribution, this framework allows the inclusion of any other background model in the computation of the null-distribution, in order to compensate for possible background bias. The probability of detecting false CISs is controlled by applying the Bonferroni correction [18], by correcting the user-defined *α*-level with the number of peaks in the resulting density estimate. This ensures that the expected error is controlled and is always smaller than the *α*-level, for any of the scales used. In contrast to previous methods, CISs can now be detected at any desirable (biologically relevant) scale, while keeping the error under control.

#### Kernel function.

Obviously, the choice of the kernel function is an important design parameter in the CIS detection. Various kernel functions (Gaussian, triangular, rectangular, Barlett-Epanechnikov, etc.) have been proposed for various applications [19]. Although in principle any type of kernel function can be used, we will compare the often-used Gaussian kernel function, the triangular kernel function, and the rectangular kernel function. The Gaussian and triangular kernel functions are “descending” kernels, which have their maximum likelihood at the observed insertion position. In addition, the Gaussian kernel is a smooth function. The rectangular kernel function possesses sharp flanks, which results in a discrete estimation of the number of insertions. Notably, the use of the rectangular kernel function bears resemblance to the approaches used in [8,9]. It is important to evaluate the performance of some kernel functions independent of any bias or noise. Therefore, we will evaluate the performance of the KC framework with artificially generated insertion data. In the text below, GKC, TKC and RKC will refer to kernel convolution using the Gaussian, triangular, and the rectangular kernel functions, respectively.

#### Scale space.

Since it is possible for CISs to be present for large scale parameters (broad CISs), but not for small scale parameters (narrow CISs), or vice versa, it is vital to consider the significance of CISs for different scale parameters. We propose a scale space approach in which the scale parameter is varied across a range of values to gain information about the “lifespan” of a CIS, while keeping the FWE at a predefined level. The lifespan is defined as the range of scale parameters for which the CIS is significant (exists). It will be shown that the CISs with a long lifespan (i.e., the CISs that appear for small as well as larger scale parameters) often consist of different narrow CISs that are joined together when increasing the scale parameter.

**Figure 3:**
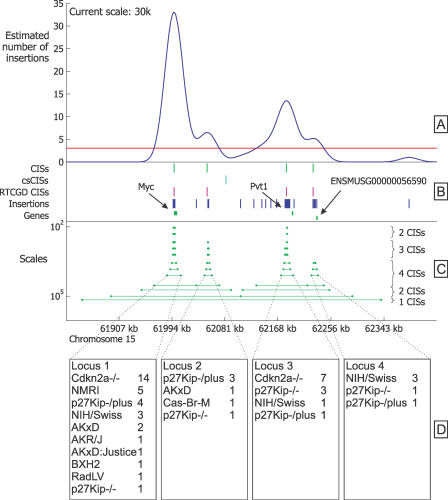
The *Myc* Locus on Chromosome 15 (A) The blue line represents the estimated number of insertions as a function of position for a certain region. The red line depicts the threshold associated with an *α*-level of 0.05. (B) CISs are depicted by means of vertical lines. From top to bottom these represent: the CISs for the current scale (30k), the csCISs, the CISs from the RTCGD, the insertions, and the genes (top and bottom strand separated). (C) Scale space diagram. The vertical axes of the scale space has a logarithmic scale and indicates the scale for which the CIS was detected (only a subset of scales was actually evaluated: [50 100 250 500 1 k 2.5 k 5 k 10 k 30 k 50 k 100 k 150 k] bp). (D) Evaluation of the insertion distribution over four small scale CISs, identified by scale space analysis. Per screen we list the number of insertions that fall within the small scale CIS. The screens are labeled consistent with RTCGD nomenclature.

Plotting the CISs versus the scale parameter yields scale space diagrams (see, e.g., Figure 3). Horizontally, the locus in the genome is plotted; vertically, the scale parameter. A CIS is represented by a horizontal line connecting the start and end positions of the CIS, defined by the intersection of the estimation of the number of peaks with the threshold. The vertical positions of these lines correspond to the scale parameter for which this CIS was found to be significant. The location of genes is also displayed in the scale space diagram, providing the opportunity to identify the genes that could be affected by a detected CIS, the relative location of CISs to genes, and the range across which a gene can be affected by a CIS. To enable comparison with the CISs from the RTCGD, we define a “cross scale CIS” (csCIS) as a unique region in the genome classified as a CIS by *at least* one scale parameter.

#### Background correction.

As mentioned before, MLV favors integration near TSS. Consequently, the location of TSS may be a good predictor for integration hot spots. We therefore explore a background model which uses the locations of the 5′ ends of the genes annotated in ENSEMBL for background bias correction. Although it has been shown that the viral insertions prefer integration near the 5′ end of *active* genes [14,16], we used all genes for our background correction, since there is no information available about which genes were active during integration. There are, however, more (unknown) factors influencing the selective behavior of MLV [16]. This makes it difficult to reliably estimate a background density, since absence of TSSs does not necessarily imply cold spots. Furthermore, the data of the RTCGD consists of insertions from screens with different types of the MLV that may prove to have slightly different preferential site selection. For these reasons, we prefer to use a background model based on both the background (TSS) data and a uniform data distribution, and hence only correct for the presence of hot spots.

### Results from Artificial Data

The performance and robustness of the KC framework in conjunction with either the Gaussian, triangular, or rectangular kernel function, is evaluated using artificial data. It consists of a uniform background distribution and one artificially generated CIS at a predefined locus. The insertions within the CIS are generated using a uniform distribution. In Figures S2 and S3 we have added the result for artificial CISs generated from a normal distribution.

The following evaluation criteria are defined (for details see the Methods section): 1) true positive (TP), the true detection of the artificially generated CIS at a significance level of 5%; 2) cross scale true positive (csTP), observing a TP for *at least* one scale parameter; 3) false positive (FP), the detection of a CIS at a location other than at the predefined locus; and 4) cross scale FP (csFP), counting an FP at a locus only once even if it occurred across multiple scales.

**Figure 4:**
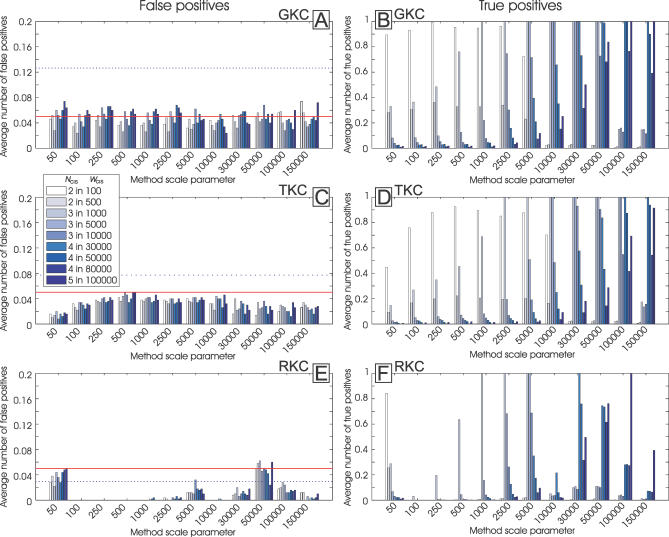
Results from Simulation Experiments—True and False Positives (A,B) Results for the GKC applied to artificial data. (C,D) Results for the TKC. (E,F) Results for the RKC. The horizontal solid lines in (A), (C), and (E) show the 5% significance level, the dotted lines show the average number of csFPs. The legend shows the different simulated CISs, stating the number of insertions *N*_CIS_ that fall within the CIS of width *W*_CIS_.

Figure 4A, 4C, and 4E clearly shows that for all the kernel functions under consideration, the error is controlled to be below the *α*-level of 5% for all the scales. The GKC controls the errors at an average of 5%, whereas the TKC manifests a slightly more conservative behavior, since the error is below 5% for all scale parameters. This becomes even more apparent for the RKC, where the errors remain well below the *α*-level for all but two scale parameters. Equally important is the fact that the error is not constant across the scales, that is, TKC and RKC suffer from a scale-dependent bias, although for the RKC this bias is far more severe than for the TKC.

This can be explained from the discrete nature of the null-distribution of peak heights (see Figure S4). Because the rectangular kernel function does not smooth insertions weighed by their distance to the observed insertion, a null-distribution of peak heights results that is discretized to an integer insertion count. A consequence of this is that, when increasing the scale parameter gradually, there will be points in the scale space for which the method suddenly becomes more conservative, because at these transition points an extra insertion is needed before significance is reached when compared with a slightly smaller scale parameter. To a lesser extent, this effect is also present for the triangular kernel function (Figure 4C). The scale-dependent bias is only truly absent when using the Gaussian kernel function, as can be seen from Figure 4A.

Thus, using the number of peaks to correct for multiple testing proves to keep the FWE below the predefined level of 5%, as can be seen from Figure 4A, 4C, and 4E. However, it is the Gaussian kernel function that is capable of controlling the error at 5%, independent of the scale. The results obtained from simulating CISs from a normal distribution also support these findings, as can be seen from Figure S2.

It should be noted that FWE is controlled per scale parameter. A range of 12 scale parameters is used so that, if for every scale parameter a *unique* FP error would occur, an average number of combined FPs of 0.6 would be expected. Plotting the average number of csFPs shows that this does not occur (Figure 4A, 4C, and 4E, dotted line). This indicates that the dependency between tests at different scales is high. For this reason, we deem extra correction for the tests over different scales unnecessary, and the resulting error acceptable.

**Figure 5:**
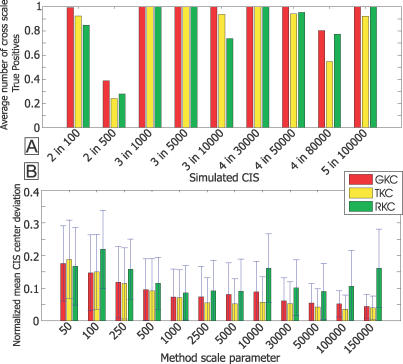
Results from Simulation Experiments—Cross Scale True Positives and Deviations from CIS Center (A) Average number of csTPs per artificially generated CIS. Significant errors are made for the borderline cases: the narrow CISs (500 bp), or broad CISs (80k bp) with relatively few insertions. The GKC outperforms the RKC and TKC for all simulated CISs. (B) Average deviation of the detected CIS center from the actual simulated CIS center normalized on the simulated CIS width plus the scale parameter under consideration.

The scale-dependent bias and consequent conservativeness of the RKC and TKC also has repercussions for the TPs (Figure 4B, 4D, and 4F). From Figure 5A we note that among the simulated CISs some borderline cases are present. The case for which only two insertions are present in a region of 500 bp is not detected with 100% accuracy. For the broad CISs with a low number of inserts, the results are similarly mediocre. This is not surprising since for those CISs, the CIS insertion rate approaches that of the background insertion rate. Still, using a triangular or rectangular kernel function seems to perform reasonably well, considering the average number of csTPs approaches one for most of the simulated CISs. However, with the FWE under control, it is the GKC that manages to reach a maximal average number of TPs for most scale parameters (Figure 4B) and most simulated CISs (Figure 5A), outperforming the TKC and RKC.

From Figure 4B, 4D, and 4F it is clear that the methods mostly reach a TP rate of one when the scale parameter is approximately equal to the simulated CIS width, indicating the specificity of the scale parameter to a certain CIS width. This specificity property is evident from the fact that small simulated CISs (light bars) are detected more frequently in the simulations using small scale parameters, whereas the large simulated CISs (dark bars) are mainly detected using larger scale parameters. Notably, the range of scale parameters across which a CIS of a certain width is detected is considerably larger for GKC and TKC than for RKC, which indicates a larger degree of robustness.

From Figure 5B it can be seen that, when considering only the TPs, the positional accuracy of the GKC and TKC are slightly better than for the RKC. This can best be explained by the fact that these kernel functions optimize the CIS location by using the peaks of the estimated number of insertions, and thereby incorporate the distribution of the insertion data in the detection of CISs, whereas the RKC only uses the two outer insertions of a CIS to position its center.

The results for a Gaussian distribution of insertions within the CIS are given in Figures S2 and S3. GKC, TKC, and RKC appear to perform only slightly worse. Still, the differences between the different kernel functions remain obvious. These results indicate that the method is relatively robust for different insertional distributions within the CIS.

In conclusion, the GKC shows a clear advantage with regard to the performance when applied to artificial data, some advantage with regard to positional accuracy, but most important, it shows a consistent error distribution across the scales. For these reasons, we propose the GKC to be the method of choice to analyze the data from the RTCGD.

### Results from RTCGD Data

#### Scale space.

Applying the GKC method to real data yields scale space diagrams, such as the one depicted in Figure 3. The same subset of scale parameters is evaluated as was used for the experiments on artificial data. The CISs are displayed in the scale space, which offers the opportunity to evaluate the lifespan of CISs across multiple scales. For instance, we learn that the locus near the gene to the right of the *Pvt1* gene is only significant for scales larger than 2.5 k bp. It is also possible that CISs are only significant for small scale parameters (see Figure S5 for a region on Chromosome 11). In the scale space diagram, we see that, for the *Myc* locus, it is equally justified to state that there is one, or that there are two, three, or four distinct CISs present, all depending on which scale range is considered.

The added value of breaking a single large-scale CIS for the *Myc* locus into a number of small-scale CISs can be illustrated by examining the genotype specificity of the CISs at a scale of 10 k bp (four CISs). Interestingly, it is observed that *Cdkn2a* null mice have a notable bias toward the first and third of these small-scale CISs. This finding suggests that inserts in these CISs functionally differ from those in the other two CISs, either in the expression levels they induce or perhaps via more complex effects on temporal regulation of *Myc* or the abundance of differentially translated *Myc* isoforms. As such these tumors might be a useful starting point to examine tumor cells' tolerance to *Myc* protein in the presence or absence of the *Cdkn2a;* for instance, with respect to *Myc-*induced apoptosis/senescence.

#### Integral analysis.

In [8] a CIS is defined as observing either two insertions within 26 k bp or three insertions within 250 k bp. When applying this definition to the 962 insertions from the combination of the screens from [8] and [7], we find 94 CISs. We can estimate the number of FPs using a permutation procedure, as detailed in [9]. This results in an estimated number of false detections equal to 11 (12%). Depending on the type of followup experiment, this might still be acceptable. The GKC detects 53 CISs, for which the probability of being a false detection is always lower than 5%.

**Figure 6:**
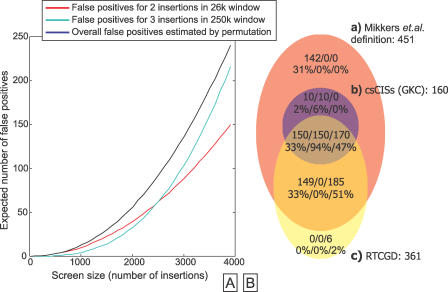
Comparison with Previous CIS Definition (A) Plot of the increase of the error as a function of the screen size, when using the definition from [8], computed using the Poisson distribution, or a permutation approach. Also the results from the two individual windows used are given. Since the errors made by the two windows individually are not mutually exclusive, the Poisson estimate is an overestimate of the true error. (B) Venn diagram comparing three different CIS definitions: a) the definition from [8] applied to the complete dataset, b) the csCISs resulting from the GKC, and c) the published CISs from the RTCGD. The intersection between sets shows three counts (and corresponding percentages), indicating the count for set a, b, and c, respectively. This is because the three sets of CISs used different definitions (at different scales) for a CIS, so that some CISs are split up, and hence are counted twice.

The limitations of the definition from [8] become evident when applying it to the complete set of data (3,947 insertions). This results in the discovery of 451 CISs, but as many as 244 (54%) are estimated to be false detections. Clearly, this definition is unsuitable for larger datasets, such as the one obtained by combining all the data from the RTCGD (also illustrated in Figure 6A). Figure 6B shows that, although our method does not find new CISs when compared with the CISs resulting from the definition in [8], our method selects the CISs that have a small chance of being false detections. In other words, the KC framework balances the number of detections and the number of false detections more efficiently by controlling the FWE. While the total number of detected CISs is reduced, the set of detected CISs is guaranteed to be TPs with a probability imposed by the *α*-level. This makes the method suitable for scaling to large datasets.

**Figure 7:**
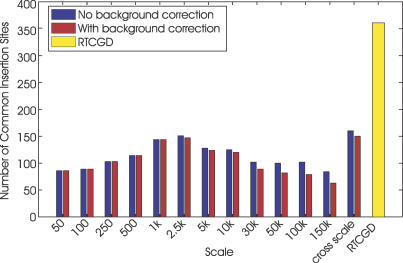
Number of CISs per Scale Parameter Number of CISs for Various Scale Parameters (Corrected and Uncorrected), the csCISs, the Background-Corrected csCISs, and the CISs from the RTCGD. Background correction only has effect at larger scales.

**Figure 8:**
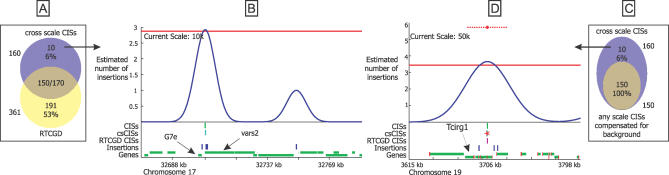
Example of Novel CIS and Background Corrected CIS (A) Venn diagram comparing the csCISs and the CISs in the RTCGD. For reasons explained in Figure 7, the intersection shows two counts. (B) An example of a CIS that consists of three insertions from three independent screens, and therefore is only detected when integrally analyzing the data. (C) Venn diagram comparing the csCISs with and without applying background correction. (D) An example of a csCIS, that was also included in the RTCGD, and is rejected based on the background-corrected threshold. The small vertical bars (red) in the genes denote the 5′ ends of genes, and a star denotes a corrected CIS. Since we are only interested in correcting regions that are putative CISs, a background-corrected threshold is only computed for peaks in the estimated number of insertions. The corrected threshold is given by the horizontal dotted line above the peak.

Figure 7 shows the number of CISs for all scale parameters individually, the number of csCISs, and the number of CISs in the RTCGD. From the Venn diagram in Figure 8A we learn that the integral analysis using the fully data-driven GKC method results in ten novel CISs (Figure 8B also shows an example) when compared with the published CISs from the RTCGD. Further analysis of the CISs indicated that six of these novel CISs could only have been discovered when integrally analyzing the data, since the individual insertions occurred in different screens. Two of the CISs consisted of two insertions either at the exact same locus or within a few base pairs (bp) of each other, in different tumors but from the same screen. For some reason these CISs have been omitted from the database by the authors of the screens. The two remaining CISs consisted of insertions occurring in the same tumor and can therefore not be called a CIS. These CISs can easily be removed by preprocessing the data and replacing insertions close together, but within one tumor, by the median insertion. Table 1 summarizes the results. We have annotated this CIS table with nearby putative target genes which might play a role in oncogenesis. Notably three of these are genes that play a role in MAP kinase signaling, whilst others have roles in Wnt signaling, lymphocyte development, and cell cycle. Several genes also show homology to known oncogenes.

**Table 1:**
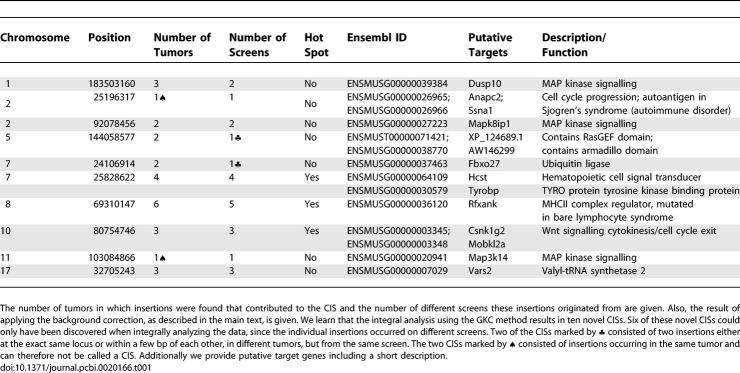
Overview of the Novel CISs Detected by GKC

As expected, the total number of detected CISs is reduced as a consequence of the control of the FWE. The discarded CISs (53%) are not necessarily all false detections; many of them may be screen-specific CISs that consisted of only few insertions and did not reach significance when we integrally analyzed the data. Also, some of the CISs in the RTCGD were found using human interpretation of the insertions. The GKC can also be applied to any relevant subset of the data, although a minimum of approximately 800 insertions is required to reliably estimate a null-distribution within a reasonable timeframe.

#### Background correction.

Additionally, the background bias was removed using the procedure described in the Methods section. Based on the results depicted in Figure 7, we can conclude that for small scale parameters no CISs were discarded. This is in accordance with the background bias model used in the analysis: a Gaussian distribution of ±5 k bp does not justify the removal of small CISs (see also Figure S6). For larger scale parameters, however, CISs exist that do not reach the background-corrected threshold for significance. When looking across the scales, the background bias accounted for a total of ten csCISs. It is important to note that three of the rejected csCISs are among the previously mentioned newly discovered CISs. When correcting for the background bias, we therefore find five novel CISs, but this is only based on the TSS as a model for the background. In Figure 8, an example of a corrected csCIS is given that would have been significant across two scales (30 k bp and 50 k bp), but did not reach the background-corrected threshold in both cases. Because we currently do not model cold spots, no novel CISs were found using the background correction.

## Discussion

Detection of CISs in large retroviral insertional mutagenesis screens at acceptable false detection rates necessitates correction for multiple testing and renders manual curation of CISs impractical. Current methods do not control the number of falsely detected CISs without changing the scale of the putative CIS, and fail when applied to large datasets. In this paper, this is solved by introducing a KC framework capable of discovering statistically significant CIS, while controlling the FWE for any biologically relevant scale. Because the KC framework controls the error per scale, it is capable of analyzing the data in the scale space, allowing the discovery of narrow as well as broad CISs.

We evaluated the performance of the KC framework using three often-used kernel functions: the Gaussian, triangular, and rectangular kernel functions. From the results obtained using artificial data, we conclude that the KC framework is capable of keeping the FWE under the desired error level, for a range of different CISs and scale parameters. The GKC, however, performs most robustly, since it is capable of controlling the error in an unbiased fashion across the scales. This is highly desirable when analyzing the data in a scale space. Additionally, the TKC and GKC show better positional accuracy when compared with the RKC.

To test the performance of the method on a large dataset, we used the GKC to integrally analyze the data from the RTCGD. This resulted in the discovery of CISs that are significant across the screens according to a consistent definition, have a low probability of being false detections, and can be analyzed in the scale space. As a consequence, 53% of the CISs previously published in the RTCGD did not reach significance in the combined dataset. Among the discovered CISs are eight novel CISs, of which six could have only been found when we integrally analyzed the data. Three of those might be attributed to the background bias, but this is based on too little evidence. For these novel CISs, the putative targets have been provided.

The KC framework is flexible enough to incorporate a background bias correction. Currently, data to base the background model on is lacking. For instance, the effect of *active* genes being favored integration targets cannot be incorporated without the data from experiments assessing which genes are active during integration. Mining data from infected cells that did not yet show proliferative selection, independent from the tumor data, and using these in the background bias correction will circumvent these problems. Still, making some assumptions about the background bias model resulted in the rejection of ten csCISs, but verification whether only true hot spots were corrected should be conducted.

As an additional benefit, the KC framework excels in visualizing the results, allowing the biologist to inspect the smoothed insertion estimate around interesting loci in the genome. Plotting the CISs in the scale space by means of scale space diagrams yield a valuable visualization tool for the biologist, showing the lifespan of CISs across a range of values of the scale parameter. This enabled the detection of screen-specific biases toward small-scale CISs. Together with the insertion locus relative to the neighboring genes, this provides useful information in determining the target of the insertional mutations.

Recently, some attention was given to multi-experiment analysis in the detection of significant copy number aberrations across experiments in array-CGH data (STAC algorithm from [20]). The STAC algorithm is designed for data containing aberrations (either deletions or amplifications) obtained by thresholding the copy number measurements. Next, it detects regions with overlap between stretches of aberrations across different samples. Although this makes it unsuitable to apply to, for instance, insertion data (since insertions only rarely exactly overlap between samples), the KC framework may be applied to copy number data. For this purpose the kernel will need to be tailored to the data type. Investigating the rules that govern the choice of the kernel function for different data types will thus further increase the usability of the KC framework as a multi-experiment analysis tool. To this end, the use of more advanced nonparametric density estimators may be investigated. Specifically the use of wavelet kernel-density estimation is of great interest.

## Materials and Methods

### Kernel convolution framework.

Convolution of the insertion data with a kernel results in a smoothed estimate of the number of insertions 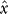 at position *g*:

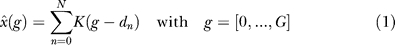
where *K*(·) is a kernel function, *d_n_* is the position of the *n* ^th^ insertion, *N* is the total number of insertions, and *G* is the genome length. Smoothing the observed insertions in a region around the position of insertion models two important phenomena. First, it models the fact that observing an insertion also contains information about its direct neighborhood. Because there is a limited number of observed insertion positions (data sparseness), it is important to exploit this information. Second, the distance across which insertions can act on a specific target gene is not fixed. There may exist multiple regions of varying widths within or around a gene for which an insertional mutation alters the function of the gene. By smoothing the observed insertions, the joint effect of the insertions on the target gene is incorporated in the model.

### Kernel function.

The design of the appropriate kernel function is important. Since we are estimating the *number* of insertions, rather than a density, we choose to normalize the kernel function such that: *K*(0) = 1, rather than the more common normalization: 
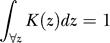
 , which produces the probability density estimate first proposed by Parzen [21]. Furthermore, in this study, we assume that there is no apparent bias to either the left or the right side of the observed insertion. This constrains the possible kernel functions to symmetrical functions.

Nondescending kernels that have sharp flanks (e.g., rectangular kernels), can only result in discrete (or even integer) estimations of the number of insertions. In this study we show that, although the error is controlled to be below the *α*-level, non-integer estimations of the number of insertions allow a less conservative and more unbiased control of the error across different scales. Nondiscrete estimations of the number of insertions can be obtained by using a descending kernel function (e.g., Gaussian, triangular kernels) that has its maximum likelihood at the observed insertion. Lastly, *smooth* kernel functions give more robust local maxima in 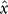 at the loci of interest, whereas nonsmooth kernels (e.g., triangular) display many noisy local maxima.

In this study the following well-known kernel functions are used and compared:

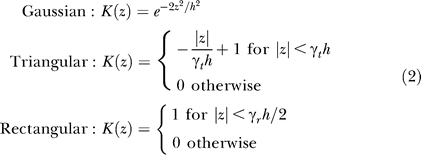
where *h* is the kernel width and *γ_t_* and *γ_r_* are normalization factors for the width of the kernel function. Because a range of scale parameters is used, the normalization of the width of the kernel functions is not crucial, as long as the range of scale parameters is chosen such that all relevant scales are covered. Here, we choose the normalization factor such that the surface under the kernel functions is equal, that is *γ_t_* = *γ_r_* = 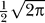 .

### Null-distribution.

**Figure 9:**
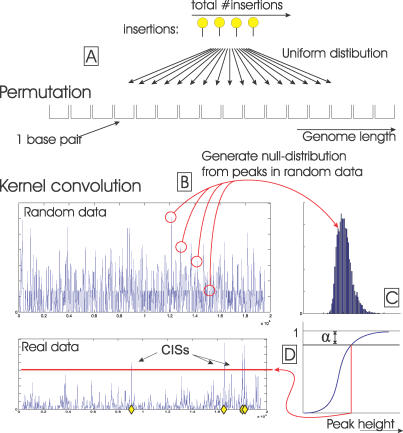
Schematic Depiction of the Significance Analysis of the Density Estimate of the Insertion Data (A) The position of the *N* insertions is permuted. (B) The convolution method is applied to the resulting permuted insertion profile. The heights of all peaks are recorded. (C) Step A and B are repeated. A distribution of the peaks in random data results. (D) The threshold is computed by determining the *α*-level in the empirical CDF of the null-distribution. This threshold is applied to the insertion estimate of the real insertion data, resulting in a series of significant peaks.

The null-distribution is estimated by a permutation-based analysis of the insertion data (see Figure 9A). More specifically, the KC is applied to a uniform random permutation of the insertion data. From this result an estimate is made of the distribution of peak heights (Figure 9B). This distribution approximates the null-distribution since it estimates the probability of finding a peak with a certain height in random data. Formally, we state the null-hypothesis as follows

**Hypothesis 1. Null-hypothesis KC**

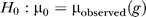
where *μ*_0_ is the mean height of the peaks in the permuted insertion data and *μ*_observed_ = 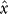 (g), the observed height of the peak. The null-hypothesis is rejected if the observed height of the peak significantly *exceeds* the mean height of the peaks in the permuted data (one-tailed test). Significance is guaranteed by thresholding the smoothed estimate of the number of insertions with an amplitude threshold for the peaks. This threshold is established by determining the peak height associated with the *α*-level in the empirical cumulative distribution function (CDF) calculated on the peaks in the permuted data (Figure 9D).

### Multiple testing correction.

The KC introduces dependencies between bp. A Bonferroni correction will therefore produce overly conservative results. When this dependence is removed by only evaluating the peaks, applying the Bonferroni correction to the *p*-values obtained for each peak can be justified. The number of tests, and hence the Bonferroni correction factor, then equals the number of peaks in the estimate.

### Cross scale CIS.

To determine the position of the csCISs, a single linkage hierarchical clustering algorithm is applied to the CIS center loci of the CISs, for all scale parameters. The resulting dendrogram is thresholded at a linkage distance equal to the highest scale parameter, to ensure good cluster separation (see Figure S7). When a CIS is detected across more than one scale, the mean of the CIS center positions is taken as the csCIS position, resulting in an estimate of the unique CIS loci across the scales.

### Background correction.

Compensating for background bias requires inclusion of local changes of the a priori insertion probability in the null-hypothesis. In the KC framework, the correction of the null-hypothesis is achieved by replacing the permutation of the insertion data with a simulation process that incorporates the background insertion distribution. Analogous to recent literature [16], we collected the 5′ end of the 27,602 genes in the Ensembl database as a model of the TSS (Genebuild: March 2005, Assembly: NCBI m34). We used a Gaussian density estimation with a kernel width of 10 k bp (which is equivalent to Gaussian distributions with a standard deviation of ± 5 k bp around the 5′ end of the genes).

**Figure 10:**
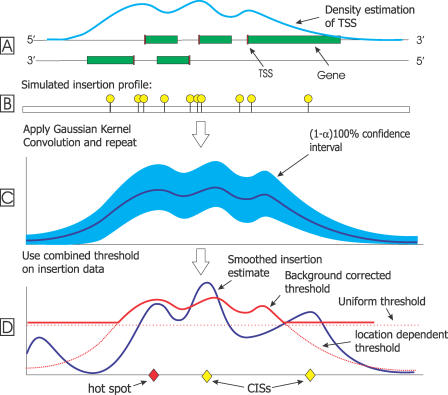
Schematic Depiction of the Computation of a Background-Corrected Threshold (A) The density of TSSs (the 5′ ends of the genes) is computed using a fixed kernel width *h*_bg_. (B) A new realization of insertions is generated using the density from step A. (C) The GKC method is applied to the resulting insertion profile, yielding one realization of the background density estimate. Steps (A) and (B) and applying the GKC are repeated N times to yield a distribution of background realizations. For every position on the genome, a CDF of these realizations is computed and the threshold is determined based on the *α*-level. (D) The location-dependent threshold is combined with the threshold based on uniform background. Finally, the smoothed insertion estimate of the real data is thresholded with the resulting threshold.

The simulation follows the steps outlined in Figure 10. First (Figure 10A), a density estimate of the TSS (with kernel width *h*_bg_) is computed. Second (Figure 10B), the simulated background data is acquired by generating a realization of the insertions according to the density estimate from step A. Third (Figure 10C), the GKC method is applied to this realization. Now, steps A and B are repeated to yield a distribution of insertion density estimates that follow the background for every location in the genome. Given this distribution of background estimates, the threshold as a function of the genome position can be obtained (Figure 10D). For clear hot spots, this threshold will be rather high, ensuring that, in the real data, a very high peak is needed before significance is reached. The null-hypothesis can now be rewritten as follows:

**Hypothesis 2. Null-hypothesis GKC corrected for background**

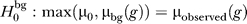
where *μ*_0_ is the mean height of the peaks in the random permutation of the insertion data, *μ*_bg_(*g*) is the mean height of the estimate of the number of insertions in the simulated background data as a function of the genome location *g*, and *μ*_observed_ (*g*) = 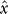 (*g*) the height of the peak in the smoothed insertion estimate. For the background-corrected significance test, both the mean height of the peaks based on uniform background as well as the mean height of the estimate of the number of insertions in background simulated data needs to be significantly *exceeded*. Significance is determined from the empirical CDF calculated on all simulation data as a function of the genome location *g*. We maintain control of the FWE by adjusting the background threshold with the same correction factor as used in the framework without background correction, since again only peaks in the insertion data are considered.

### Artificial data experiments.

To simulate the background, a uniform distribution of *N*_artificial_ = 400 insertions is generated on an artificial genome of *G*_artificial_ = 2.6 × 10^8^ bp long. The CIS is generated by drawing *N*_CIS_ insertions from a uniform distribution centered at 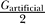 and bounded by 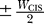 .

The width and number of insertions of the CIS is varied to evaluate the performance of the method for different types of CISs. *W*_CIS_ is varied from 100 bp to 100 k bp, covering the relevant CIS widths. *N*_CIS_ is chosen such that the methods operate at a critical point for which some errors are made, enabling good comparison (for the exact parameters see the legend in Figure 4 or Table S1). For each experiment, 500 artificial datasets were generated to simulate the effect of having different insertion distributions in the background and within the CIS. For every artificially generated dataset, the methods were employed for the following scale parameters: [50 100 250 500 1 k 2.5 k 5 k 10 k 30 k 50 k 100 k 150 k] bp. A significance level of *α* = 5% was chosen.

To evaluate the accuracy and performance of the methods, some criteria are defined. Using artificial data allows us to evaluate the correctness of a detected CIS, since the actual CIS locus is known. A TP is defined as the true detection of the artificially generated CIS, and is accomplished if the method identifies a CIS that has one of its bounds within the bounds of the artificially generated CIS (given by *W*_CIS_). Additionally, we define the csTPs as observing a TP for *at least* one scale parameter (or equivalently: the detection of a csCIS).

An FP is defined as the detection of a CIS that does not pass the test for a TP, and hence occurs in the background. It should be noted that the probability of making at least one FP (the FWE) is controlled per scale parameter. If the errors made per scale parameter were mutually exclusive, this could result in an undesirably high overall error. To analyze this behavior, the average number of csFPs is computed, which counts an FP at a locus only once even if it occurred across multiple scales.

The average number of TPs and FPs are computed by taking the mean across the 500 simulations. Since FPs only occur in the background, distinguishing between the different simulated artificial CISs makes no sense. Therefore, the average number of csFPs is computed by additionally averaging across all different experiments.

As a final performance measure, the positional accuracy is evaluated. For this purpose, the deviation of the detected CIS center with respect to the artificial CIS center is normalized to the artificial CIS width plus the scale parameter *W*_CIS_ + *h*. If the artificial CIS is detected more than once, the mean of the CIS center positions is taken as the detected position.

## Supporting Information

Figure S1 Schematic—and Idealized—Overview of the Tumor Development Process and the Procedure to Acquire the Insertion Loci (A) The red insertions are oncogenic insertions, i.e., insertions either activating oncogenes or inactivating tumor suppressor genes, causing uncontrolled proliferation (cell division) of that cell, yielding a tumor. The green insertions are non-oncogenic insertions. Note that in every tumor cell we find this red insertion at exactly the same locus, since these cells are copies.
(B) After removal of the tumor from the animal, the tumor cells' DNA is cleaved into small fragments using restriction enzymes. These enzymes cut the DNA at certain nucleotide sequences, resulting in small DNA fragments. An additional property of these enzymes is that they cut exactly within the viral inserts, yielding fragments with viral DNA on one end and host cells' DNA on the other.
(C) The histogram shows the abundance of fragments. Because the restriction enzymes cut frequently, many fragments result. However, while only a limited number of insertions is present in each cell, by far most fragments do not contain an insertion. Due to the proliferation in (A), the fragments with oncogenic inserts will be present more often than the fragments containing the non-oncogenic (random) insertions. Note that in reality these abundances are not known.
(D) With a polymerase chain reaction (PCR), only the fragments containing a viral insert are amplified (multiplied). There exist various types of PCR reaction that may be used for this purpose, i.e., the linker-mediated PCR [22] or the inverse PCR (IPCR) [23 and references herein]. In the first place, this results in removal of fragments not containing a viral insert, and second, enough material is generated for the sequencing.
(E) Shows the histogram of the abundances of DNA fragments after the PCR. After PCR amplification, the oncogenic (red) insertions will be more prevalent than others, and should all map to exactly the same locus on the genome (defined as a contig). Due to noise in the following steps (sequencing and mapping), this may differ by several hundred bp. Singletons are sequences that individually map to a certain locus on the genome and ideally consist of the non-oncogenic insertions.
(F) Finally, a subset of the fragments is cloned, sequenced, and mapped onto the known Ensembl mouse genome. It is highly probable that an informative (oncogenic) insertion is sequenced and mapped, because the abundance of DNA fragments containing oncogenic insertions is a substantial proportion of the total number of insertions. Thus it might occur that a non-oncogenic insertion is sequenced and mapped, hence the data contains a certain amount of noise.
(294 KB EPS)

Figure S2 Results for Simulating CISs from a Normal Distribution (A,B) Results for the GKC applied to artificial data.
(C,D) Results for the TKC.
(E,F) Results for the RKC.
The horizontal solid lines in (A), (C), and (E) show the 5% significance threshold, the dotted lines show the average number of csFPs. The legend shows the different simulated CISs, stating the number of insertions drawn from a normal distribution of standard deviation: *σ*_CIS_.
(871 KB EPS)

Figure S3 Results for Simulating CISs from a Normal Distribution (A) Average number of csTPs per artificially generated CIS. Only the CIS with three insertions with *σ* = 1 k bp reaches 100% detection. For all other CISs, some errors are made. The GKC outperforms the RKC and TKC for all simulated CISs.
(B) Average deviation of the detected CIS center from the actual simulated CIS center normalized on the simulated CIS width plus the scale parameter under consideration.
(380 KB EPS)

Figure S4 Depiction of the Null-Distributions The threshold corrected for multiple testing is given by the horizontal red line. Note that the threshold should change because when the scale parameter increases the number of tests (the number of peaks) decreases. This is not visible in Figure S4. From these figures it becomes clear that when increasing the scale parameters there are transitions (from 50 to 100, 5 k to 10 k, 50 k to 100 k, and 100 k to 150 k bp) where an extra insertion is needed before significance is reached. These transitions can also be clearly identified in Figures S2 and S3.
(608 KB EPS)

Figure S5 Estimated Number of Insertions, CISs, and Scale Space Diagram for a Locus on Chromosome 11 The blue line represents the estimation of the number of insertions as a function of position for a certain region. The red line depicts the 0.05 threshold level. In the middle, the CISs are depicted by means of vertical lines. From top to bottom these represent: the CISs for the current scale (30k, green), the csCISs (cyan), the CISs from the RTCGD (magenta), and the insertions (blue). The genes are not shown on this large scale. At the bottom, the CISs are plotted in the scale space. The vertical axis has a logarithmic scale and indicates the scale for which the CIS was detected (only a subset of scales was actually evaluated: [50 100 250 500 1 k 2.5 k 5 k 10 k 30 k 50 k 100 k 150 k] bp).
(547 KB EPS)

Figure S6 Number of CISs That Fall within One Scale Parameter from a TSS CISs that fall within one scale parameter from a TSS are candidates for correction. We clearly see that for small scale parameters only a few of these CISs exist, indicating that it is not justified to correct narrow CISs for background bias.
(254 KB EPS)

Figure S7 Schematic Depiction of the Clustering Process of the CIS Centers across the Scales to acquire the csCISs A single linkage dendrogram is built from the CIS centers, and thresholded at a linkage distance equal to the highest scale parameter. The mean center position of the CISs within one of the resulting clusters is defined at the locus of the csCIS. Note that a csCIS arises if a CIS is present for at least one scale parameter. In case a CIS is present for only one scale parameter, the csCIS locus is equal to the CIS center position.
(239 KB EPS)

Table S1 Overview of the Artificial Data Experiment Settings *N*_CIS_ denotes the number of inserts in an artificially generated CIS of width *W*_CIS_, in bp. The rate is defined as *N*_CIS_ / *W*_CIS_.
(45 KB DOC)

## Abbreviations

CIS: common insertion site
csCIS: cross scale CIS
csFP: cross scale false positive
FWE: family-wise error
FP: false positive
GKC: Gaussian kernel convolution
KC: kernel convolution
MLV: murine leukemia virus
PCR: polymerase chain reaction
RKC: rectangular kernel convolution
RTCGD: Retroviral Tagged Cancer Gene Database
TKC: triangular kernel convolution
TP: true positive
TSS: transcription start site
γ_r_: normalization factor for rectangular kernel
γ_t_: normalization factor for triangular kernel
μ_0_: mean height of the peaks in the permuted insertion data
μ_bg_: mean height of the estimation of the number of insertions in the permuted insertion data
μ_observed_: observed height of a peak
*d*_n_: position of the n^th^ insertion
*g*: base pair position in the genome
G: genome length in base pair
*G*_artificial_: genome length in artificial data experiment
*h*: kernel width
*h*_bg_: kernel width used for density estimation of the background
K(·): kernel function
*N*: total number of insertions
*N*_artificial_: number of insertions in artificial data experiment
*N*_CIS_: number of insertions within artificial data experiment
smoothed estimate of the number of insertions
*W*_CIS_: width of the CIS in artificial data experiment
