# Supplementary material for: Detecting Statistically Significant Common Insertion Sites in Retroviral Insertional Mutagenesis Screens
Source: PLoS Comput Biol. 2006 Dec 8;2(12):e166. doi: 10.1371/journal.pcbi.0020166 (PMC1676030; doi:10.1371/journal.pcbi.0020166)
Supplement: Supplementary file 1 [file pcbi.0020166.st001.doc]

| Experiment |  |  | Rate |
| --- | --- | --- | --- |
| 1 | 2 | 100 |  |
| 2 | 2 | 500 |  |
| 3 | 3 | 1k |  |
| 4 | 3 | 5k |  |
| 5 | 3 | 10k |  |
| 6 | 4 | 30k |  |
| 7 | 4 | 50k |  |
| 8 | 4 | 80k |  |
| 9 | 5 | 100k |  |
